# A Metadata description of the data in "A metabolomic comparison of urinary changes in type 2 diabetes in mouse, rat, and human."

**DOI:** 10.1186/1756-0500-4-272

**Published:** 2011-07-29

**Authors:** Julian L Griffin, Helen J Atherton, Christoph Steinbeck, Reza M Salek

**Affiliations:** 1 MRC Human Nutrition Research, Elsie Widdowson Laboratory, Fulbourn Road, Cambridge, CB1 9NL, UK; 2 The Department of Biochemistry, Tennis Court Road, University of Cambridge, Cambridge CB2 1GA, UK; 3 The Cambridge Systems Biology Centre, Tennis Court Road, University of Cambridge, Cambridge CB2 1GA, UK; 4 The MRC Centre for Obesity and Related Diseases (MRC CORD), the University of Cambridge Metabolic Research Laboratories, Addenbrooke's Hospital, Cambridge CB2 0QQ, UK; 5 The European Bioinformatics Institute, Wellcome Trust Genome Campus, Hinxton, Cambridgeshire CB10 1SD, UK

**Keywords:** data standards, metabolomics repository, bioinformatics, NMR spectroscopy

## Abstract

**Background:**

Metabolomics is a rapidly developing functional genomic tool that has a wide range of applications in diverse fields in biology and medicine. However, unlike transcriptomics and proteomics there is currently no central repository for the depositing of data despite efforts by the Metabolomics Standard Initiative (MSI) to develop a standardised description of a metabolomic experiment.

**Findings:**

In this manuscript we describe how the MSI description has been applied to a published dataset involving the identification of cross-species metabolic biomarkers associated with type II diabetes. The study describes sample collection of urine from mice, rats and human volunteers, and the subsequent acquisition of data by high resolution 1H NMR spectroscopy. The metadata is described to demonstrate how the MSI descriptions could be applied in a manuscript and the spectra have also been made available for the mouse and rat studies to allow others to process the data.

**Conclusions:**

The intention of this manuscript is to stimulate discussion as to whether the MSI description is sufficient to describe the metadata associated with metabolomic experiments and encourage others to make their data available to other researchers.

## Background

Metabolomics as a functional genomic tool is rapidly growing in popularity for a range of applications across all the kingdoms of life. This is both being driven and driving developments in analytical chemistry, particularly NMR spectroscopy and mass spectrometry, to increase the capabilities of measuring metabolites in biofluids, tissues, cell culture media and even whole organisms. A diverse range of approaches are also applied across metabolomics including open profiling approaches aimed at detecting as wide a range of metabolites as possible, commonly used in biomarker discovery and functional genomic studies, or closed profiling where metabolites of a given class are targeted, with this approach being more amenable to quantification as well as lending itself to hypothesis directed research.

However, one limiting factor for the future development of metabolomics is data exchange. In a recent prospective note two of the authors of this article observed: "there is still a very small number of actual studies that make their data available, and even fewer in a format that would comply with the Metabolomics Standards Initiative (MSI) description [1]." The MSI set out to define the minimum information that is required to capture the necessary metadata to describe a metabolomic experiment, in much the same way as initiatives such as MIAME for microarrays and MIAPE for proteomics [2-4]. In any such initiative there are obvious tensions between the desire to completely describe an experiment and still make the description user friendly to ensure experimentalists will use it. The recommendations of MSI produced a number of publications to describe this minimum information [5-8].

The aim of this report is to describe how one might report this minimum metadata alongside the raw and processed data of a previously published study. Our aims are two-fold. Firstly, by demonstrating how this data should be reported we hope to encourage others to make their data available for the wider scientific community. Ultimately we hope to stimulate the creation of dedicated databases for metabolomic data to allow others to cross compare results from multiple studies. This may in turn have significant savings financially and, for mammalian work, reduce the total number of animals required for future studies. An illustrative example from our own area of research is in the use of metabolomics as a functional genomic tool in obesity and diabetes research. New mouse models which are thought to suffer from diabetes are often compared with results from known models, particularly the dbdb and obob mouse strains where leptin signalling is impaired [9,10]. Because there is no recognised database for metabolomic data it is often necessary to include a cohort of mice in the study of a known phenotype to cross-compare with. This is both costly and animal intensive. Metabolomic databases will allow the storage of previous results and ultimately allow comparison across even more models. Secondly, by making the raw and processed data available we also hope to aid bioinformaticians involved in the development of new processing and statistical tools.

The study we have chosen is a published study of two rodent models of type II diabetes and human sufferers of the disease [11]. For each species high resolution ^1^H NMR spectroscopy was used to profile the metabolic composition of urine, and then through a combination of principal components analysis (PCA) and partial least squares discriminate analysis (PLS-DA) metabolites distinct to each model and also common across all species were identified.

## Evidence of use

The metadata description is based on the descriptions developed under MSI [5-8].

## Metadata description

The publication Salek et al., 2007 [11] in fact consists of three separate studies: two of rodent models of type II diabetes and one study of human sufferers of type II diabetes. Thus, to describe the metadata of the paper there are three supplementary files dealing with the description of the individual studies [Additional files 1, 2 and 3]. The format of the metadata follows the description used by Fiehn and co-workers in [12] and we thank Prof. Fiehn for making the Excel spreadsheet available for use here. Considering the mouse data, the metadata file starts by describing the animals, and in particular what gene modification has been performed, what tissue or biofluid is analyzed and how much material is collected during the study. For strain and genotype of animals the recommendation is to use the recognized convention if available for that species. For mice we have used the strain description used by JAX laboratories http://jaxmice.jax.org/strain/000642.html.

This is then followed by a brief description of the animal housing, diet and water. Given the obvious impact diet has on the metabolome it is particularly important to describe this factor as the phenotype of a mouse model of diabetes can vary markedly depending on whether the mice are on a carbohydrate diet, as in this study, or on a high fat diet, which increases the severity of many aspects of the metabolic syndrome. This information is relatively straight forward to collect for most laboratory animal studies but may not be available for human studies or environmental studies where the subjects are free living. Under experimental design the groups used for comparisons are described. Most studies will have a relatively simple description of animal numbers used in a study but for reference [11] sample collection was performed on three genotypes, both genders and either as part of 24 or 48 hr sample collections. As a result a supplementary table was required to capture this information. Note also for the manuscript the heterozygous and wildtype control mice were treated as a single class and so the numbers are reported in the same manner here.

Next, information is captured concerning sample extraction. For a biofluid study this is relatively brief and usually captures how the sample was diluted down, but for tissues this part of the metadata would capture what extraction procedure was used. In this study the sample is diluted in phosphate buffer used to ensure the pH is maintained at 7.2 and hence avoid shifts of key resonances associated with the variability of pH of the collected urine. The sample also has D_2_O added as a lock reference, sodium azide as a preservative and sodium 3-trimethylsilyl-(2,2,3,3-^2^H_4_)-1-propionate (TSP) as a chemical shift reference.

No information is required in this study under chromatography, but information is then recorded for NMR spectroscopy. This would be replaced by mass spectrometry descriptions if the study had used this particular approach. For NMR spectroscopy it is particularly important to capture information concerning the pulse sequence used to acquire spectra. In the current study a commonly used solvent suppression pulse sequence was used, but for intact tissue and blood plasma/serum markedly different results could be obtained depending on whether the pulse sequence is edited for T_1 _or T_2 _relaxation or diffusion properties of the metabolites present. Furthermore, the description of the pulse sequence also allows the reader to judge whether the spectra are acquired under fully relaxed conditions or under semi-saturated conditions which has important consequences for subsequent quantification. In order for people to complete the analysis performed in the paper it is also necessary to report how the data was subsequently processed both in terms of how the raw data was converted into a format suitable for statistical analysis, and how the subsequent statistical analysis is performed. To allow others to re-create the analysis in [11], as well as develop new tools for the processing of NMR based metabolomic studies the supplementary data include the original spectra for the mouse and rat studies and the normalised integral files used in the pattern recognition models [Additional files 4, 5, 6, 7, 8, 9 and 10]. For the normalised integral files the glucose region has been excluded - this is because in the original paper the aim was to identify potentially new markers of type 2 diabetes, and not the obvious one of increased glucose excretion! The excluded integral regions were 3.22-3.30, 3.38-3.58, 3.70-3.94, 5.22-5.28 ppm [Additional files 1, 2, 4].

The deposition of NMR spectra raises the issue as to what format spectra should be made available in. Although JCAMP is a fairly uniformly accepted data standard for NMR spectroscopy it is so rarely used and most programs will readily process all vendor formats, that we have chosen to use the vendor's format - in this case that used by Bruker BioSpin. Another issue is the potential misuse of data which is made available to the wider community. As the human data formed part of an on-going series of drug trials we are not in a position to make this data available to the wider community, and thus any initiative to make data available in the metabolomic community must consider that parts of the community may not be able to make all of their data publically available.

This study involved both data from laboratory animals and humans. The human study had a number of challenges to capture key metadata in terms of the study design. While genotype was not an appropriate category for a free living study where no sequencing data was collected an important component of the study was the inclusion and exclusion criteria of the patients. This has been included as free text in the metadata.

One caveat with this report is the standards for metabolomics are still evolving and although there are descriptions of what is desired in a metadata description of a metabolomics experiment, there is no consensus across the community. However, only by beginning to use the recommendations can experimentalists get a feel for whether the descriptions capture enough or too much information.

## Carrots rather than sticks?

The biggest problem with data standards and making data available to the community is the extra work required by the experimentalist to make that data available. A description of the metadata involved in an experiment will always take some extra time during the submission process of a paper or a final report for a grant. One way to ensure complicity is to go down the route of 'sticks' and mandate scientists to submit data as part of the manuscript submission process, as occurs already for many microarray studies, or when a final report for a grant is submitted. However, it's also important to consider the carrots associated with making data available. Firstly, it encourages others to develop tools for the datasets that are deposited. Secondly, it also encourages others to reference the work. However, we feel the major carrot is the ability for groups to work across multiple sites. If we consider metabolomics in functional genomics, the ultimate aim is to understand how the modification of every gene influences the metabolism of the organism being studied. This is an immense challenge that no one laboratory could hope to achieve. The ability to develop databases for specific organisms and disease processes will allow multiple labs to work together and store their data alongside one another. These on-line resources will become vital research tools for the community in much the same way GenBank has supported gene sequencing and the Gene Expression Omnibus (GEO) and the Microarray Gene Expression Database (MGED) has supported the microarray community.

## Abbreviations

GEO: Gene Expression Omnibus; JCAMP: Joint Committee on Atomic and Molecular Physical Data; MGED: Microarray Gene Expression Database; MIAME: Minimum Information About a Microarray Experiment; MIAPE: The minimum information about a proteomic experiment; MSI: Metabolomics Standards Initiative; NMR: nuclear magnetic resonance.

## Competing interests

JG and CS are recipients of a BBSRC grant entitled MetaboLights to develop a central repository and curated resource for metabolomic data.

## Authors' contributions

JLG conceived and wrote the manuscript, and took part in the original study. HJA contributed to the writing of the manuscript. CS contributed to the writing of the manuscript and the description of metadata. RMS contributed to the writing of the manuscript and took part in the original study. All authors read and approved the final manuscript.

## Supplementary Material

Additional file 1 **Supplementary_data_human**. Metadata associated with the human diabetes study. Click here for file

Additional file 2 **Supplementary_data_mouse**. Metadata associated with dbdb mouse diabetes study. Click here for file

Additional file 3 **Supplementary_data_rat**. Click here for file

Additional file 4 **Rar file containing all the spectra from the dbdb mouse diabetes study in Bruker format**. Click here for file

Additional file 5 **Rar file containing all the spectra from the Zucker rat diabetes study in Bruker format**. Click here for file

Additional file 6 **Rar file containing all the spectra from the Zucker rat diabetes study in Bruker Format**. Click here for file

Additional file 7 **Rar file containing all the spectra from the Zucker rat diabetes study in Bruker format**. Click here for file

Additional file 8 **Excel spread sheet containing normalised integral files excluding the glucose region generated from the human diabetes study**. Click here for file

Additional file 9 **Excel spread sheet containing normalised integral files excluding the glucose region generated from the dbdb mouse diabetes study**. Click here for file

Additional file 10 **Excel spread sheet containing normalised integral files excluding the glucose region generated from the Zucker rat diabetes study**. Click here for file
